# Invasive species removal increases species and phylogenetic diversity of wetland plant communities

**DOI:** 10.1002/ece3.5188

**Published:** 2019-04-23

**Authors:** Shane C. Lishawa, Beth A. Lawrence, Dennis A. Albert, Daniel J. Larkin, Nancy C. Tuchman

**Affiliations:** ^1^ Institute of Environmental Sustainability Loyola University Chicago Chicago Illinois; ^2^ Department of Natural Resources and the Environment and Center for Environmental Science and Engineering University of Connecticut Storrs Connecticut; ^3^ Department of Horticulture Oregon State University Corvallis Oregon; ^4^ Department of Fisheries, Wildlife and Conservation Biology and Minnesota Aquatic Invasive Species Research Center University of Minnesota St. Paul Minnesota

**Keywords:** biological invasions, ecological restoration, Great Lakes, *Typha*, wetlands

## Abstract

Plant invasions result in biodiversity losses and altered ecological functions, though quantifying loss of multiple ecosystem functions presents a research challenge. Plant phylogenetic diversity correlates with a range of ecosystem functions and can be used as a proxy for ecosystem multifunctionality. Laurentian Great Lakes coastal wetlands are ideal systems for testing invasive species management effects because they support diverse biological communities, provide numerous ecosystem services, and are increasingly dominated by invasive macrophytes. Invasive cattails are among the most widespread and abundant of these taxa. We conducted a three‐year study in two Great Lakes wetlands, testing the effects of a gradient of cattail removal intensities (mowing, harvest, complete biomass removal) within two vegetation zones (emergent marsh and wet meadow) on plant taxonomic and phylogenetic diversity. To evaluate native plant recovery potential, we paired this with a seed bank emergence study that quantified diversity metrics in each zone under experimentally manipulated hydroperiods. Pretreatment, we found that wetland zones had distinct plant community composition. Wet meadow seed banks had greater taxonomic and phylogenetic diversity than emergent marsh seed banks, and high‐water treatments tended to inhibit diversity by reducing germination. Aboveground harvesting of cattails and their litter increased phylogenetic diversity and species richness in both zones, more than doubling richness compared to unmanipulated controls. In the wet meadow, harvesting shifted the community toward an early successional state, favoring seed bank germination from early seral species, whereas emergent marsh complete removal treatments shifted the community toward an aquatic condition, favoring floating‐leaved plants. Removing cattails and their litter increased taxonomic and phylogenetic diversity across water levels, a key environmental gradient, thereby potentially increasing the multifunctionality of these ecosystems. Killing invasive wetland macrophytes but leaving their biomass in situ does not address their underlying mechanism of dominance and is less effective than more intensive treatments that also remove their litter.

## 1 INTRODUCTION

Plant invasions have been linked to losses in biodiversity (Gaertner, Breeyen, Hui, & Richardson, 2009; Powell, Chase, & Knight, 2011; Vilà et al., 2011) and changes in ecosystem functions, including nutrient and carbon regulation (Ehrenfeld, 2003; Liao et al., 2008) and soil microbial processes (Hawkes, Wren, Herman, & Firestone, 2005). However, quantifying changes in ecosystem function, correctly attributing changes to invasive plants, and disentangling the effects of anthropogenic ecosystem degradation from invasive plant‐driven changes (MacDougall & Turkington, 2005) can be difficult. Plant phylogenetic diversity integrates across many plant traits and ecological differences and correlates with key ecosystem functions (Srivastava, Cadotte, MacDonald, Marushia, & Mirotchnick, 2012), including community productivity (Cadotte, Cavender‐Bares, Tilman, & Oakley, 2009) and community stability (Cadotte, Dinnage, & Tilman, 2012). Thus, phylogenetic diversity can be used as a readily quantifiable metric for predicting multifunctionality of ecosystems, and when combined with traditional plant diversity analyses, results in a broader assessment of ecological conditions. As such, restoration efforts increasingly consider analyses of phylogenetic diversity (Barak et al., 2017; Barber et al., 2017; Larkin et al., 2015), though this practice is not yet widespread.

Laurentian Great Lakes (GL) coastal wetlands are well‐suited to test questions about the effects of invasive plants and their restoration on phylogenetic diversity and taxonomic diversity due to both their functional importance and increasing dominance by invasive macrophytes (Carson et al., 2018). GL coastal wetlands provide regionally critical habitat for diverse plant communities (Albert & Minc, 2004), fish (Uzarski, Burton, Cooper, Ingram, & Timmermans, 2005), and migratory waterfowl (Prince, Padding, & Knapton, 1992), and key ecosystem services (Sierszen, Morrice, Trebitz, & Hoffman, 2012). Water‐level fluctuations occurring at multiyear to decadal time scales are the primary natural disturbance in GL coastal wetland ecosystems (Minc, 1997; Trebitz, 2006) and are largely responsible for maintaining high plant diversity by stimulating recruitment and establishment (Wilcox, 2004; Wilcox & Nichols, 2008). Over the short term (1–3 years), individual plant species respond uniquely to water‐level changes (Gathman, Albert, & Burton, 2005) and over the longer term, the breadth of wetland plant zones expand and contract following fluctuations (Frieswyk & Zedler, 2007; Minc, 1997). Both high‐ and low‐water events tend to reset successional trajectories (Wilcox, 2004). Directly following water‐level retreat, mudflat conditions become common, creating ideal conditions for plant germination from persistent sediment seed banks (Keddy & Reznicek, 1986) and sprouting from semidormant rhizomatous perennials (Albert, Cox, Lemein, & Yoon, 2013).

Invasive plants, namely cattail (hybrid cattail: *Typha *× *glauca*; narrowleaf cattail: *T. angustifolia*; hereafter *Typha*) and European common reed (*Phragmites australis* ssp. *australis*), have proliferated over recent decades in these ecosystems (Carson et al., 2018; Trebitz & Taylor, 2007; Tulbure & Johnston, 2010). Range expansions by *Typha* and *P*. *australis* have been associated with establishment during low‐water conditions in the 2000s (Lishawa, Albert, & Tuchman, 2010; Tulbure & Johnston, 2010). Invasive *Typha* tolerates a wide range of water levels (Harris & Marshall, 1963), invades across the hydrologic gradient, and once established, tends to become highly dominant (Lishawa et al., 2010). Invaded wetlands exhibit reduced plant diversity and altered ecosystem conditions compared to uninvaded sites (Lishawa et al., 2010; Tuchman et al., 2009). Experiments have demonstrated that steadily accumulating and slowly decomposing leaf litter is a principal factor responsible for loss of native plant species from *Typha‐*invaded wetlands (Larkin, Freyman, Lishawa, Geddes, & Tuchman, 2012).

Although it is clear that invasive *Typha* is correlated with reduced site‐level emergent plant diversity (Boers, Veltman, & Zedler, 2007; Galatowitsch, Anderson, & Ascher, 1999; Lishawa et al., 2010; Tuchman et al., 2009), and the accumulation of litter is a primary driver of this diversity loss (Larkin et al., 2012; Vaccaro, Bedford, & Johnston, 2009), it is unclear how restoration treatments targeting various intensities of litter removal will affect native plant communities and their phylogenetic diversity. During a period of GL‐wide low‐water levels, when the potential for seed bank driven regeneration of native plants was highest, we tested plant community and phylogenetic diversity responses within two wetland zones to treatments comprising a gradient of *Typha* removal intensities: control (no manipulation), mow (cutting and leaving aboveground biomass in situ to kill stems without addressing legacy litter), harvest (aboveground biomass and litter removal to kill stems and remove legacy litter), and complete (belowground and aboveground biomass and litter). In order to evaluate the potential for plants to regenerate from the seed bank following *Typha* removal across the range of naturally occurring water levels in GL wetlands, we paired the field experiment with a seed‐bank study investigating seedling emergence within each zone under three water levels. We hypothesized that (H1) *Typha* removal should result in seed bank germination stimulated diversity increases and (H2) these responses should increase with increasing *Typha* removal intensity.

## 2 MATERIALS AND METHODS

### 2.1 Study site

**Figure 1:**
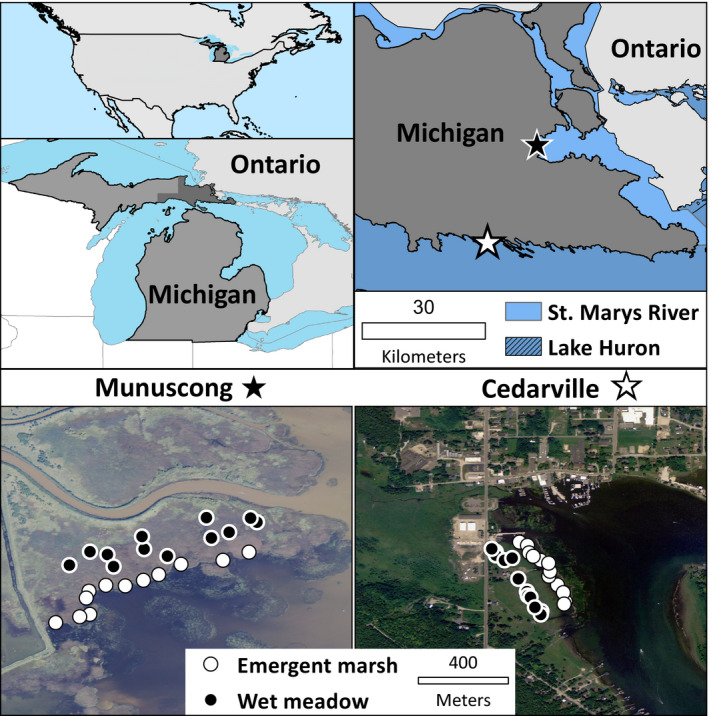
Maps of study locations and aerial imagery of Cedarville and Munuscong Marshes showing plot layout within the two marsh zones

We conducted our experiment in Cedarville Marsh and Munuscong Marsh, two invasive *Typha*‐dominated wetlands in northern Michigan (USA; Figure 1). Both sites are GL‐connected and exposed to the long‐term water‐level fluctuations within the GL system; at the time of the study (2011–2013), water levels in the GLs were at the end of a 13‐year low‐water period (Gronewold, Clites, Smith, & Hunter, 2013). Cedarville Marsh is a GL lacustrine protected‐embayment wetland (Albert, Wilcox, Ingram, & Thompson, 2005) in Cedarville, Michigan (lat 45.99282 N, long 84.36039 W), disturbed by urban development and nutrient enrichment from wastewater treatment effluent. Munuscong Marsh is a GL‐connecting channel river delta wetland (Albert et al., 2005) on the St. Marys River (lat 46.20435 N, long 84.25201 W), which connects Lakes Superior and Huron. Munuscong Marsh has been degraded by diking for wildlife management and nutrient enrichment from agricultural runoff.

### 2.2 Field experiment

During 2011–2013, we implemented a vegetation‐manipulation experiment testing the effects of marsh zone (two levels) and *Typha* removal intensities (four levels) in two wetlands (two levels). Within *Typha*‐dominated areas (>50% relative dominance) of each marsh zone, we randomly located 12, 16‐m^2^ plots (4 × 4 m) using the *Generate Random Points* tool in ET Geo Wizards (Tchoukanski, 2008) in ArcMap (Environmental Systems Research Institute). In the wet meadow zones, we randomly assigned three treatments (harvest, mow, and control) × four replicates. Within the emergent marsh zone, we randomly assigned four treatments (complete removal, harvest, mow, and control) × three replicates. We established plots in July 2011 and implemented treatments in August 2011. Complete removal consisted of cutting all stems at the sediment surface using an aquatic weed whacker (Weeders Digest LLC), removing all aboveground biomass and litter from the plot, and hand‐harvesting all rhizomes from the sediment (complete rhizome removal involved substantial time and effort, requiring as much as 20 person‐hours per‐plot); harvesting involved cutting all stems at the sediment surface and removing biomass and litter from the plot; mowing involved cutting all stems at the sediment surface and leaving biomass in situ. We did not implement complete removal treatments in the wet meadow because it was infeasible due to deep rooting and highly organic soils. To isolate our treatment areas and prevent translocation of nutrients and carbohydrates from outside plots, in 2011 and 2012, we severed belowground connections along all plot perimeters by cutting through roots and rhizomes using an ice chopper, a heavy‐duty sharpened metal blade attached to a wooden pole. With enough downward force, the chopper traveled through the organic layer to the mineral sediment, severing all rhizomes. Within each 16‐m^2^ plot, we established four 1‐m^2^ subplots located 0.5 m from the perimeter at plot corners.

In late‐July of each year (2011, 2012, 2013), we sampled the vegetation in each subplot by assigning areal cover values (<1%–100%) for each plant species, total vegetative cover, and litter. We recorded the presence of additional plant species within the larger 16‐m^2^ plots, by systematically scanning the plot periphery following completion of subplot data collection. Total species richness in the plot and the mean cover values of the four subplots were used for analysis.

### 2.3 Seed bank experiment

We used the seedling emergence method (Davis & Van der Valk, 1978) to test emergent marsh versus wet meadow seed bank responses to water‐level manipulations. In July 2011, we collected three arbitrarily located 5‐cm deep sediment plugs with a bulb planter from each 16‐m^2^ field plot and composited these three subsamples. Sediment samples were cold‐stratified by storing them at 4°C from July 2011 to June 2012 when the experiment began. We removed detritus, rhizomes, and roots and then composited within‐zone samples and thoroughly homogenized the sediments by hand. We spread a 1‐cm thick subsample of homogenized sediment over the surface 9.5‐cm‐diameter pots (70.9‐cm^3 ^sediment per/pot) filled to the depth of 10 cm with autoclave‐sterilized sand. We randomly assigned pots to three different water‐level treatments (relative to soil surface): high (+5 cm), moist (0 cm), or low (−5 cm). Four replicates of each zone × water‐level treatment were tested (2 sites × 2 zones × 3 water levels × 4 replicates = 48 total). In June 2012, pots were placed randomly within an environmental growth chamber under a fluctuating light and temperature regime approximating June conditions in the GL region: 16‐hr light at 22.5°C and eight‐hour dark at 12.5°C (Lawrence, Fahey, & Zedler, 2013). Throughout the 6‐month study period, we maintained water levels twice per week. Every 2 weeks, we re‐randomized pot locations and identified and counted seedlings. Positively identified seedlings were removed from the pots, and unidentified seedlings were allowed to grow until identification to species (or for one taxon, only to genus) was possible. All plant taxonomy followed Voss and Reznicek (2012).

### 2.4 Phylogeny construction and diversity measures

We used a published tree (Zanne et al., 2014) of over 32,000 plant taxa to construct community phylogenies of the 142 taxa identified in our field study and 26 species identified in our seed‐bank study. Nonangiosperm taxa (*n* = 9) were excluded from our analyses. Species that were not included in the Zanne *et al.* tree were placed in the tree at the crowns of their respective genera. We calculated plot‐level phylogenetic diversity using abundance‐weighted forms of Faith's phylogenetic diversity (PD), mean pairwise phylogenetic distance (MPD), and imbalances of abundance of higher clades (IAC) (Cadotte et al., 2010; Faith, 1992; Webb, Ackerly, McPeek, & Donoghue, 2002). These metrics represent richness, divergence, and regularity (evenness) measures of phylogenetic diversity, respectively, that is, the total evolutionary history found in a community, how closely related co‐occurring species are, and how evenly evolutionary history is distributed among species (Tucker et al., 2017). These measures of phylogenetic diversity have been shown to be positively correlated with key ecosystem functions (Cadotte et al., 2012; Srivastava et al., 2012).

### 2.5 Data analyses

We used our seed‐bank data to test the effects of zone (emergent, meadow) and water depth on seed‐bank seedling density, species richness, and phylogenetic diversity (PD, MPD, and IAC) using linear mixed effects models with site as a source of random error. Because underlying wetland conditions (e.g., hydrology and soil mineral content) and plant communities differ substantially between zones (Minc, 1997), we analyzed zones independently in both the seed bank and field experiments. To analyze the field experiment data, we used linear mixed effects models with site as a source of random error to evaluate the effects of treatment and year on plant community metrics, phylogenetic diversity metrics, and environmental variables (*Typha* cover [%], total litter [%], total vegetation cover [%], species richness, PD, MPD, and IAC) and change in variables between pre‐ and post‐treatment. We used the *lme* function in the nlme package in R (Pinheiro et al., 2017) and assessed differences between treatments within years using the least squared means approach and Tukey's HSD. We analyzed seed bank and field‐measured multivariate plant community composition and structure using nonmetric multidimensional scaling (NMDS) with post hoc vector analysis to evaluate the correspondence between environmental variables and community structure. To assess differences between plant community groups (zone, site, zone × site), we used permutational multivariate analysis of variance (PERMANOVA) using the *adonis* function to test for differences in multivariate community structure (Anderson, 2001; Anderson & Walsh, 2013). To evaluate correspondence between plant species, treatment, and marsh zone, we used indicator species analysis (Dufrêne & Legendre, 1997); indicator values of plant species were tested via Monte Carlo simulation using 1,000 permutations. All statistical analyses were conducted using R 3.4.2 (R Core Team, 2017), with the vegan package used for NMDS and *adonis* (Oksanen et al., 2018), the indicspecies package for indicator species analysis (Dufrêne & Legendre, 1997), and the picante and pez packages for phylogenetic analyses (Kembel et al., 2010; Pearse et al., 2015). All means are presented ±1 *SE*.

## 3 RESULTS

### 3.1 Variation in pretreatment plant communities

**Figure 2:**
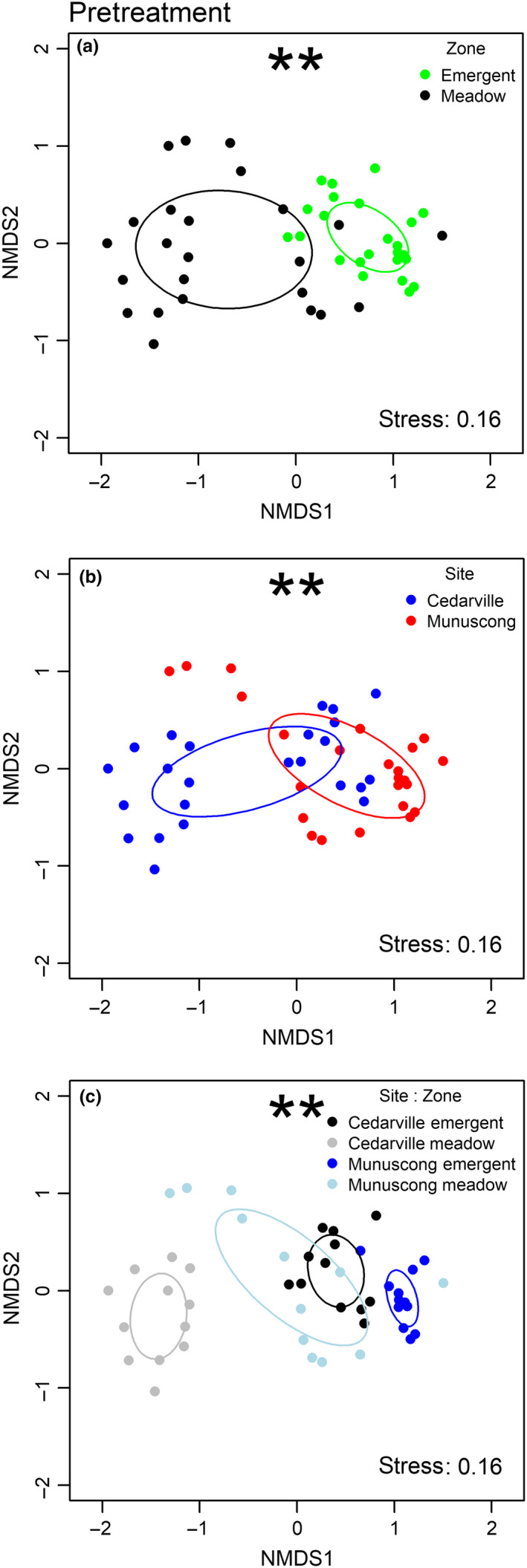
Nonmetric multidimensional scaling ordination plots of the pretreatment plant communities at two Great Lakes coastal wetlands. Points are labeled based on a posteriori group classification: (a) plant communities (emergent marsh, wet meadow), (b) site (Cedarville, Munuscong), (c) site × community. Ellipses represent one standard deviation around the centroid of each group. Significant differences between groups determined by permutational multivariate analysis of variance (PERMANOVA). **p < 0.01

*Typha* cover (%) was greater in the emergent marsh (35.01 ± 3.78) than the wet meadow (17.89 ± 2.03; *p* = 0.007), although species richness did not differ between zones (meadow: 13.37 ± 1.06; emergent: 11.17 ± 0.61 species/plot; *p* = 0.13). The pretreatment emergent marsh and wet meadow plant communities clearly diverged in multivariate species space (Figure 2a) and PERMANOVA revealed significant differences between the structure of emergent and wet meadow zones (*F* = 8.02, *p* < 0.01), between sites (*F* = 8.69, *p* < 0.01), and between site × zone (*F* = 8.55, *p* < 0.01; Figure 2).

### 3.2 Variation in seed banks.

**Figure 3:**
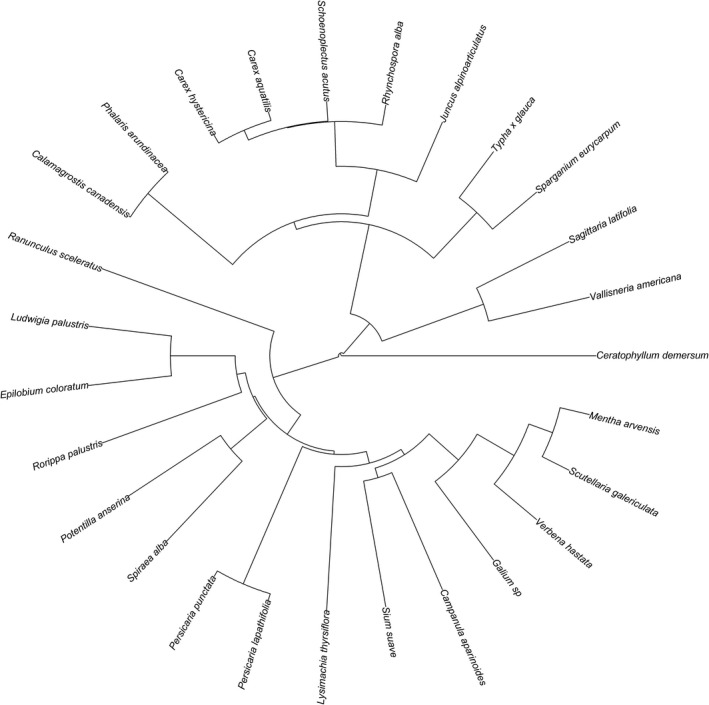
A phylogeny of all plant taxa (*n* = 26) found in the soil seed bank experiment

We generated a phylogeny of all plant taxa (*n* = 26) found in the soil seed bank experiment (Figure 3). Wet meadow seed banks had significantly greater species richness, PD, MPD, and IAC than those of emergent marsh seed banks across all water levels. In the wet meadow, water treatment was a significant factor in nearly all tested variables; high‐water treatments had reduced richness, seedling density, PD, and IAC compared to moist and low‐water treatments; however, MPD did not vary by water level. In the emergent marsh, seedling density was greater in the low‐water treatment than the high‐water treatment, but no other variables differed by water‐level treatment (Table 1).

**Table 1:** Results of a linear mixed effects models (with site as a random effect) evaluating the effects of water treatment (high: +5 cm; moist: 0 cm; low: −5 cm) on seed‐bank plant and phylogenetic diversity within the emergent marsh and wet meadow zones

Variable	Water level	Emergent marsh		Wet meadow	
		Fixed effects estimate ± *SE*	*p*	Fixed effects estimate ± *SE*	*p*
Log species richness	Moist	−0.10 ± 0.24	NS	0.46 ± 0.21	*
	Low	−0.10 ± 0.24	NS	0.74 ± 0.21	**
Log seedling density	Moist	0.11 ± 0.39	NS	1.16 ± 0.15	***
	Low	0.91 ± 0.39	*	1.76 ± 0.15	***
PD	Moist	25.50 ± 65.52	NS	264.68 ± 69.53	**
	Low	25.33 ± 65.52	NS	16.15 ± 69.53	*
MPD	Moist	−13.78 ± 21.36	NS	78.28 ± 38.58	NS
	Low	−12.98 ± 21.36	NS	37.05 ± 38.58	NS
IAC	Moist	0.03 ± 0.02	NS	0.07 ± 0.01	***
	Low	0.03 ± 0.02	NS	0.05 ± 0.01	***

Note

Fixed effects estimates are compared to the high‐water (+5 cm) treatment.

NS: *p* > 0.05.

* *p* < 0.05.

** *p* < 0.01.

*** *p* < 0.001.

### 3.3 Restoration response

**Figure 4:**
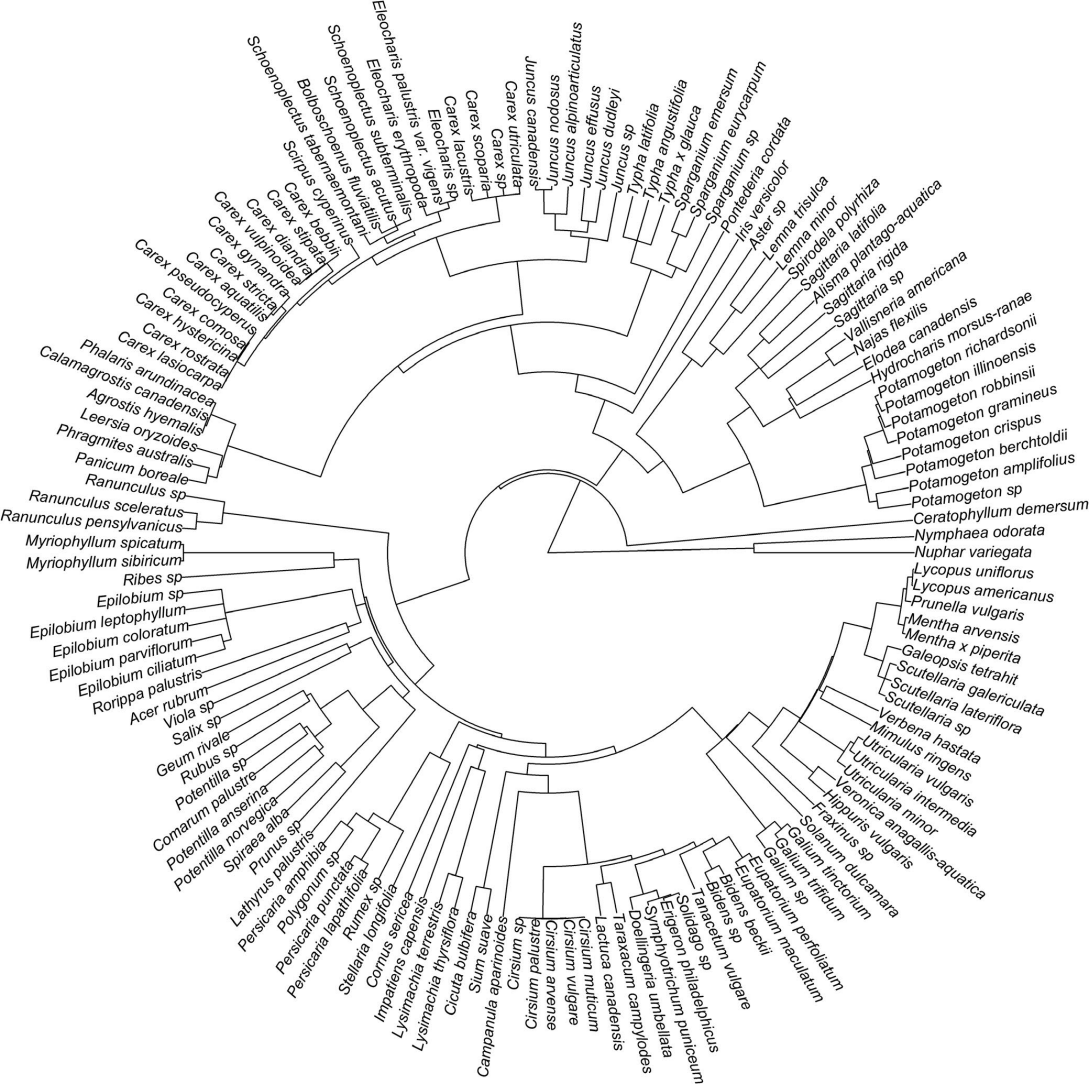
A phylogeny of all plant taxa (*n* = 142) found in 2011–2013 field restoration experiment

**Figure 5:**
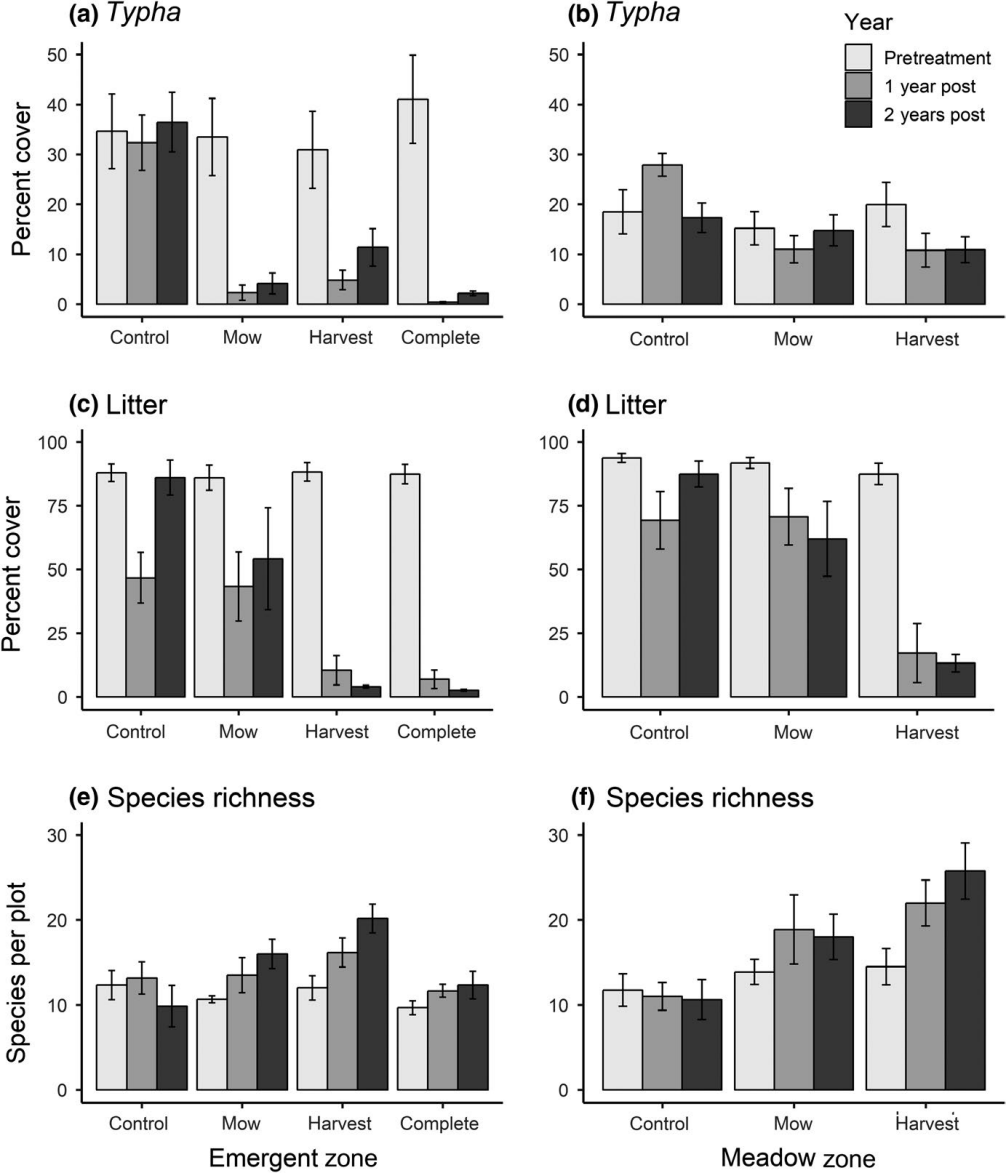
Treatment effects (mean ± *SE*) on three primary response variables, *Typha* (% cover; a,b), litter (% cover; c,d), and species richness (spp. / 16‐m^2^ plot; E, F) compared to untreated controls within the emergent marsh and wet meadow zones

**Figure 6:**
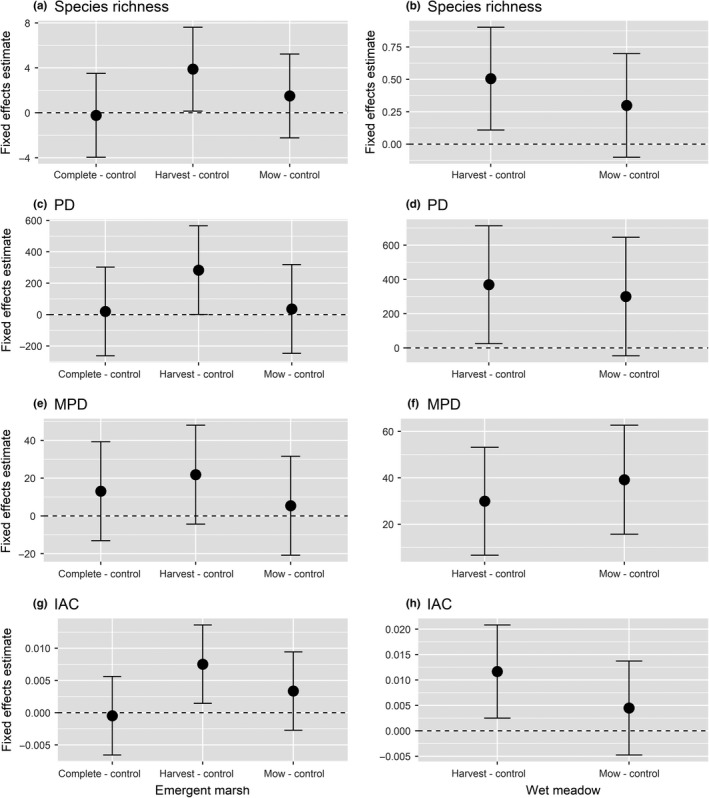
Treatment effects (±95% CI) on measures of taxonomic and phylogenetic diversity: species richness (a,b), Faith's phylogenetic diversity (PD; b,c), mean pairwise phylogenetic distance (MPD; e,f), and imbalances of abundance of higher clades (IAC; g,h) compared to untreated controls within the emergent marsh and wet meadow zones. Treatment effects were significant (*p* < 0.05) relative to controls where error bars do not overlap 0

**Figure 7:**
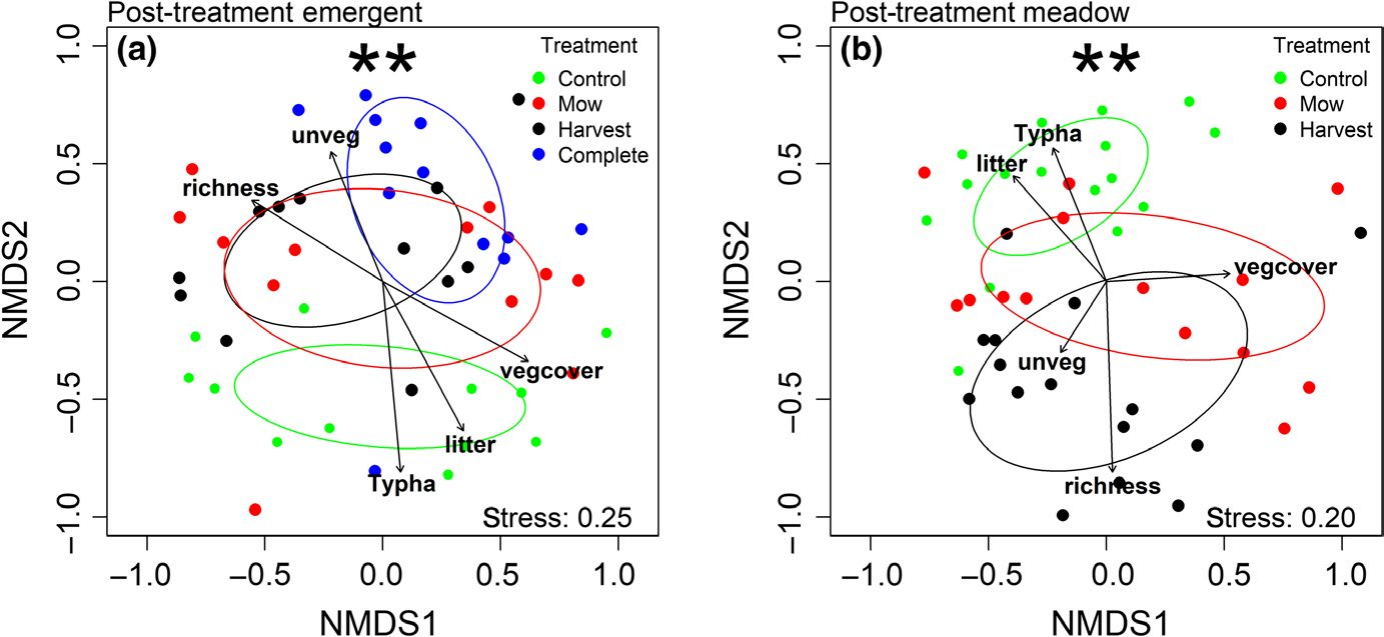
Nonmetric multidimensional scaling ordination plots of the post‐treatment plant communities at two Great Lakes coastal wetlands labeled by treatment within: (a) emergent marsh, (b) wet meadow. Fitted vector arrows are significant (*p* < 0.05, by permutation procedure), and their length is proportional to their explanatory strength: litter = litter cover (%); richness = plant species richness; *Typha* = *Typha* cover (%); unveg = total unvegetated cover (%); vegcover = total green vegetation cover (%). Differences between treatments determined by permutational multivariate analysis of variance (PERMANOVA). **p < 0.01

We generated a phylogeny of all plant taxa (*n* = 142) found in the field restoration experiment (Figure 4). *Typha* removal treatments altered measured environmental and diversity metrics in each wetland zone. *Typha* cover was affected by treatment, year, and treatment × year in the emergent marsh, and all three treatments (complete, mow, and harvest) reduced *Typha* cover relative to the control (Table 2; Figure 5). In contrast, there was only a marginally significant effect of treatment on *Typha* cover in the wet meadow zone (*p* < 0.10). Harvesting resulted in increased plant species richness in both zones (Figure 6a‐b), whereas complete removal in the emergent marsh and mowing in both zones had no effects relative to controls. Two years following treatment, harvest plots had significantly greater species richness (23.4 ± 2.1 species/16‐m^2^ plot) than mow plots (17.1 ± 1.5) and more than double the species found in control treatments (10.3 ± 1.6). Harvesting reduced litter in both zones, and complete removal reduced litter in the emergent marsh, but mowing did not affect litter in either zone compared to controls (Appendix 1 Table 2). Post‐treatment taxonomic diversity (richness) decreased linearly with increasing litter cover in both zones (emergent marsh: *R* ^2^ = 0.11, *p* < 0.05; wet meadow: *R* ^2^ = 0.15, *p* < 0.01). Treatments significantly altered the multivariate community structure in both the emergent marsh and the wet meadow zones (PERMANOVA: emergent marsh *F* = 2.67, *p* < 0.01; wet meadow *F* = 2.48, *p* < 0.01; Figure 7). In the emergent zone, pairwise PERMANOVA tests revealed that complete, harvest, and mow treatments all differed from controls (*F* = 5.33, *p* = 0.01; *F* = 2.64, *p* < 0.05; *F* = 3.08, *p* < 0.05, respectively); complete removal differed from both the harvest (*F* = 1.86, *p* < 0.05) and mow treatments (*F* = 2.19, *p* < 0.05), but harvest did not differ statistically from mow (*F* = 1.08, *p* = 0.36). Treatments similarly resulted in divergent plant communities in the meadow zone, where harvest and mow communities differed from controls (*F* = 6.53, *p* < 0.05; *F* = 2.65, *p* < 0.05; Figure 7b), whereas harvest and mow communities did not differ (*F* = 1.30, *p* = 0.22). The effect of treatments on PD and IAC reflected richness in both wetland zones, with harvesting increasing PD and IAC, but mowing and complete harvest having no significant effect relative to controls. MPD did not differ between treatments in the emergent marsh, whereas in the wet meadow, MPD was greater in both the harvest and mow treatments than in the controls (Figure 6).

**Table 2:** Results of a linear mixed effects models (with site as a random effect) evaluating the effects of treatment and year on plant and phylogenetic diversity within the emergent marsh and wet meadow zones

Variable	Factor	Emergent marsh			Wet meadow		
		*df*	*F*	*p*	*df*	*F*	*p*
*Typha* cover (%)	Treatment	3	8.30	***	2	3.30	•
	Year	2	56.68	***	2	0.56	NS
	Treatment × Year	6	7.35	***	4	2.60	•
Litter cover (%)	Treatment	3	16.24	***	2	18.57	***
	Year	2	77.58	***	2	26.46	***
	Treatment × Year	6	9.02	***	4	7.05	***
Vegetation cover (%)	Treatment	3	0.24	NS	2	1.51	NS
	Year	2	1.58	NS	2	8.07	**
	Treatment × Year	6	0.48	NS	4	0.34	NS
Species richness	Treatment	3	3.26	*	2	5.78	*
	Year	2	3.93	*	2	4.57	*
	Treatment × Year	6	3.05	*	4	4.30	**
PD	Treatment	3	2.97	*	2	3.49	*
	Year	2	0.20	NS	2	2.07	NS
	Treatment × Year	6	1.19	NS	4	1.54	NS
MPD	Treatment	3	0.21	NS	2	8.28	**
	Year	2	2.62	•	2	1.54	NS
	Treatment × Year	6	0.61	NS	4	0.12	NS
IAC	Treatment	3	5.20	**	2	4.52	*
	Year	2	2.41	NS	2	0.99	NS
	Treatment × Year	6	1.70	NS	4	1.00	NS

Note

NS: *p* > 0.10.

• *p* < 0.10.

* *p* < 0.05.

** *p* < 0.01.

*** *p* < 0.00.

In the wet meadow, two graminoids (*Juncus nodosus* and *J. alpinoarticulatus*) had significant (*p* < 0.05) fidelity to harvest, a forb and a graminoid (*Galium trifidum* and *Carex hystericina,* respectively) were associated with harvest and mow treatments, and one shade‐tolerant forb was associated with the control treatment (*Impatiens capensis*). In the emergent marsh, one emergent forb (*Sagittaria latifolia*) was associated with the harvest treatment, one submergent species (*Potomogeton richardsonii*) was associated with harvest and complete removal treatments, one graminoid (*Calamagrostis canadensis*) was associated with harvest and mow treatments, and three submergent species were associated with complete, harvest, and mow treatments (Table 3).

**Table 3:** Results of indicator species analysis of plant data by wetland zone in 2013, 2 years following treatment

Treatment	Species	Type	Indicator value	*p*
Wet meadow				
Harvest	*Juncus nodosus*	Graminoid	0.84	**
	*J. alpinoarticulatus*	Graminoid	0.67	*
Control	*Impatiens capensis*	Forb	0.92	*
Harvest + Mow	*Galium trifidum*	Forb	0.87	*
	*Carex hystericina*	Graminoid	0.77	*
Emergent marsh				
Harvest	*Sagittaria latifolia*	Forb	0.84	*
Harvest + Complete removal	*Potamogeton richardsonii*	Submergent	0.79	*
Harvest + Mow	*Calamagrostis canadensis*	Graminoid	0.69	*
Complete removal + Harvest + Mow	*Myriophyllum sibiricum*	Submergent	1.00	**
	*Utricularia vulgaris*	Submergent	0.91	**
	*U. minor*	Submergent	0.82	*

Note

Indicator value represents the proportion of perfect indication within a treatment or combination of treatments.

* *p* < 0.05.

** *p* < 0.01.

## 4 DISCUSSION

### 4.1 Diversity differed by wetland zone

Prior to treatments, wet meadow seed banks were richer in phylogenetic and taxonomic diversity than the emergent marsh, likely resulting from the emergent marsh's harsh physical conditions unsuitable for the persistence of many seeds and short‐statured plants, namely a thin organic layer (<5 cm) over mineral sediments, persistent standing water, wave action, winter ice scour, and open lake exposure (Albert et al., 2005; Minc, 1997). Seed bank germination from wet meadow soils exhibited reduced phylogenetic and taxonomic diversity under the high‐water treatment, reflecting the critical importance of moist (not flooded) soil conditions for wet meadow seed bank germination (Keddy & Reznicek, 1986), whereas emergent zone germination did not tend to differ by water‐level treatment. Our data illustrate that seed bank emergence tests are likely to underestimate diversity, yet the seed‐bank species composition clearly reflects wetland communities and the potential for management to restore these communities. In the field, in contrast with diversity measures, *Typha* had significantly greater cover in the emergent zone pretreatment, which may have resulted from water levels; during the study period (2011–2013), GL water levels were so low (Gronewold et al., 2013) that water tables in both wet meadows were below the sediment surface. Taken together, the consistent differences in measured variables supported our decision to analyze treatment responses within each zone independently.

### 4.2 Taxonomic and phylogenetic diversity responses to restoration treatments

Aboveground harvesting of *Typha* biomass and its litter increased plant taxonomic and phylogenetic diversity across both wetland zones, and richness was negatively correlated with litter cover. This positive relationship between harvesting and diversity occurred independent of *Typha* cover; in the wet meadow, litter was strongly reduced by treatment but *Typha* cover was only marginally reduced. Surprisingly, mowing had no significant effect on diversity metrics, implicating litter as the dominant factor responsible for *Typha*'s plant community impacts, which is consistent with previous research in GL wetlands (Larkin et al., 2012; Vaccaro et al., 2009). Dense litter limits seed bank germination by reducing heat and light penetration (Grime et al., 1981; Kettenring, Gardner, & Galatowitsch, 2006; Larkin et al., 2012; Lawrence, Lishawa, Rodriguez, & Tuchman, 2016; Lishawa, Lawrence, Albert, & Tuchman, 2015), and its removal creates conditions more conducive for germination (Lishawa et al., 2015). This effect was further illustrated by the response of *Juncus nodosus* and *J. alpinoarticulatus*, which were indicator species significantly associated with harvesting in wet meadow plots. *Juncus* spp. are prolific in GL coastal wetland seed banks (Keddy & Reznicek, 1986), and these early seral species can become the dominant emergent plants following water‐level reduction and mudflat exposure (Tuchman unpublished), but they quickly disappear from the emergent community with succession or *Typha* invasion (Larkin et al., 2012; Tuchman et al., 2009). In the emergent marsh, cutting biomass below standing water (all treatments) effectively reduced *Typha* abundance, likely by preventing aeration and causing rhizome mortality (Jordan & Whigham, 1988; Murkin & Ward, 1980). While complete removal in the emergent zone did not increase diversity metrics, it clearly shifted the plant community; four species of submergent or floating plants (*Utricularia vulgaris*, *Lemna minor*, *L. trisulca*, and *Potamogeton richardsonii*) were significantly associated with this treatment. Similarly, Lishawa et al. (2017) found that below‐water cutting increased submergent plant cover while reducing diversity and emergent plant cover. Complete biomass removal in the meadow zone was impractical in this study; however, previous work showed increases in taxonomic diversity when *Typha* was completely harvested under nonsubmerged conditions (Lishawa et al., 2015).

Our data revealed that harvesting *Typha* biomass and its litter increased taxonomic and phylogenetic diversity in both GL emergent marshes and wet meadows. These results provide further support for the hypothesis that *Typha* and its litter drive species loss from these ecosystems, rather than simply correlating with anthropogenic disturbance, and add to the body of evidence that *Typha* specifically, and invasive plants in general, are capable of driving species loss (Gaertner et al., 2009; Hall & Zedler, 2010; Larkin et al., 2012; Lishawa et al., 2010; Mitchell et al., 2011; Powell et al., 2011; Tuchman et al., 2009; Vilà et al., 2011). Furthermore, the increase in phylogenetic diversity indicates potential for corresponding increases in a variety of ecosystem functions that tend to be positively associated with phylogenetic diversity (Cadotte et al., 2009, 2012; Srivastava et al., 2012). It is important to recognize, however, that invasive species’ impacts on diversity are largely scale‐dependent (Powell et al., 2011; Powell, Chase, & Knight, 2013), and while the diversity recovery that we observed was apparent at the scale of our study (stratified random sample of ~38 ha of wetland), our data do not allow us to determine whether *Typha* is causing species loss or extirpation at a regional scale.

### 4.3 Implications for management

Under uninvaded conditions, widely fluctuating GL water levels over decadal time scales periodically create early successional conditions that stimulate seed bank germination (Keddy & Reznicek, 1986) and the proliferation of species with a long‐term persistent rhizomatous habit (Albert unpublished). However, more than 35% of all GL coastal wetlands are now dominated by three highly productive invasive plant taxa: *Typha* spp., *Phragmites australis*, and *Phalaris arundinacea* (Carson et al., 2018). These species produce copious and persistent leaf litter, which reduces plant diversity and creates physical conditions unsuitable for germination, even under low‐water conditions. Our study demonstrates that invasive *Typha* removal treatments (i.e., biomass harvesting and litter removal) both stimulate increased phylogenetic and taxonomic diversity and reduce *Typha* dominance in GL wetlands across wetland zones during a low‐water period, adding further support to the growing body of work that demonstrates similar diversity responses to removal across a wide range of *Typha* stand ages and water levels (Lishawa et al., 2017, 2015). In contrast, we found that a one‐time, mowing treatment without associated biomass removal reduced *Typha* dominance in the flooded emergent marsh but was ineffective at increasing plant diversity. Following biomass removal, periodic treatments would be necessary to maintain diversity and prevent the re‐establishment and dominance of invasive species over the long term. Restoration techniques that fail to address the underlying mechanisms that lead to invasive plant dominance (e.g., litter accumulation) will be ineffective at creating conditions favorable for native species regeneration.

## CONFLICT OF INTEREST

None declared.

## AUTHOR CONTRIBUTIONS

S.L., B.L, D.A., and N.T. conceived the ideas and designed methodology. S.L., B.L, and D.A. implemented the treatments and collected the data. S.L. and D.L. analyzed the data. S.L. led the writing of the manuscript. All authors contributed critically to the drafts and gave final approval for publication.

## Figures and Tables

**Figure A1:**
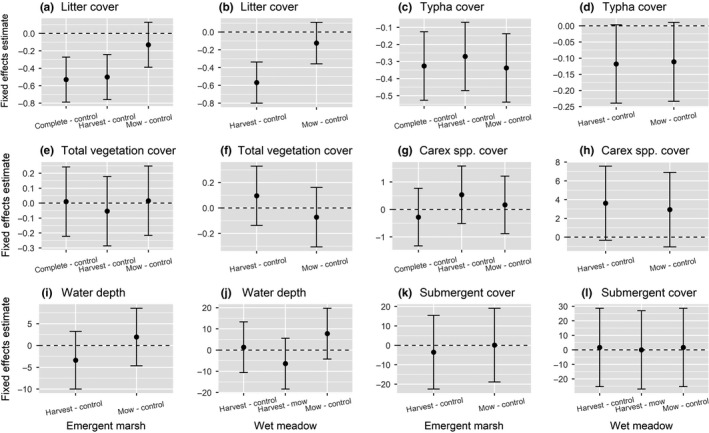
Treatment effects (±95% CI) on measures of environmental conditions and plant cover in the emergent marsh and wet meadow zones: litter cover (a,b), *Typha* cover (c,d), total vegetation cover (e,f), *Carex* spp. cover (g,h), water depth (i,j), submergent plant cover (k,l). Treatment effects were significant (*p* < 0.05) relative to controls where error bars do not overlap 0

## Data Availability

The plant community, environmental, taxonomic diversity, and phylogenetic diversity data from the field and seed bank emergence experiments are available from the Dryad Digital Repository at https://doi.org/10.5061/dryad.32h05c2.
